# Label-free and label-based electrochemical detection of disease biomarker proteins

**DOI:** 10.5599/admet.2162

**Published:** 2024-05-11

**Authors:** Tias Febriana Hanifa Lestari, Irkham Irkham, Uji Pratomo, Shabarni Gaffar, Salma Nur Zakiyyah, Isnaini Rahmawati, Seda Nur Topkaya, Yeni Wahyuni Hartati

**Affiliations:** 1 Department of Chemistry, Faculty of Mathematics and Natural Sciences, Universitas Padjadjaran, 45363, Indonesia; 2 Department of Chemistry, Faculty of Mathematics and Natural Sciences, Universitas Indonesia, 16424, Indonesia; 3 Department of Analytical Chemistry, Faculty of Pharmacy, Izmir Katip Celebi University, Turkey

**Keywords:** Biosensor, biomarker, electrochemical detection, protein detection

## Abstract

**Introduction:**

Biosensors, analytical devices integrating biological sensing elements with physicochemical transducers, have gained prominence as rapid and convenient tools for monitoring human health status using biochemical analytes. Due to its cost-effectiveness, simplicity, portability, and user-friendliness, electrochemical detection has emerged as a widely adopted method in biosensor applications. Crucially, biosensors enable early disease diagnosis by detecting protein biomarkers associated with various conditions. These biomarkers offer an objective indication of medical conditions that can be accurately observed from outside the patient.

**Method:**

This review comprehensively documents both label-free and labelled detection methods in electrochemical biosensor techniques. Label-free detection mechanisms elicit response signals upon analyte molecule binding to the sensor surface, while labelled detection employs molecular labels such as enzymes, nanoparticles, and fluorescent tags.

**Conclusion:**

The selection between label-free and labelled detection methods depends on various factors, including the biomolecular compound used, analyte type and biological binding site, biosensor design, sample volume, operational costs, analysis time, and desired detection limit. Focusing on the past six years, this review highlights the application of label-free and labelled electrochemical biosensors for detecting protein biomarkers of diseases.

## Introduction

A biosensor is a chemical sensor that uses the recognition properties of biological compounds in a sensitive layer [1]. Biosensors are analytical devices that combine sensing elements of biological compounds (enzymes, antibodies, proteins, nucleic acids, tissues or receptors, and cells) and are closely related to physiochemical transducers. Biosensors consist of three main interconnected components, *i.e.*, (1) bioreceptors or biological recognition systems, (2) transducers, and (3) electronic devices. A biological recognition system provides a sensor with high selectivity to the analyte being measured. The basic principle of biosensors is the recognition of biological compounds and sensing so that when a biological compound is recognized by a recognition compound, a signal change occurs by the transducer [2-7]. The key to a biosensor device is the transducer used. Transducers take advantage of the physical changes that accompany the reaction. The working principle of a biosensor based on the transducer used is divided into calorimetric biosensors (based on heat released or absorbed by the reaction), electrochemical biosensors (based on changes in voltage, current, or conductance), piezoelectric biosensors (based on the change of mass of products or reactants), optical biosensors (based on light output or differences in light absorbance of a product or reactant) [8]. Electrochemical transducers are widely used in point-of-care testing because they are simple, portable, cost-effective, and easy to use [8].

Detection of disease biomarkers, such as proteins or metabolites in human body fluids, is one of the diagnostic applications [9]. Biomarker detection is increasingly in demand due to its high demand in various fields, such as biotechnology, health care and life sciences [10]. Currently, there is a pressing need for monitoring human health status by analyzing biochemical markers such as glucose, galactose, cholesterol, uric acid, and urea. In recent years, biosensors have been widely used as a fast and convenient alternative to conventional analytical methods, which play a role in monitoring human health status with biochemical analytes [11]. The utilization of biosensors for disease detection, particularly in the case of cancer, is extensive due to their exceptional performance and real-time detection capabilities. Furthermore, biosensors possess a notably low minimum detection limit, enabling the measurement of biomarkers at extremely low levels in physiological samples for early-stage disease diagnosis. Additionally, biosensors can concurrently detect multiple biomarkers [12]. Notably, biosensors also offer the advantage of simultaneous detection of multiple biomarkers, presenting a versatile and efficient approach to disease diagnosis.

Several review articles have discussed electrochemical biosensors for various disease biomarkers that have been reported. However, no one has discussed the details of the electrochemical label-free or labeled detection mechanism. This review covers protein biomarkers of diseases, label-free and labeled biosensors detection techniques, and the utilization of electrochemical biosensors for detecting disease-related protein biomarkers over the past six years to help other researchers in developing biosensors. Furthermore, the future perspective was also discussed.

## Electrochemical biosensors

Electrochemical measurements are based on electrochemical processes or changes in electrical signals that occur due to electrochemical reactions on the electrode surface. The reaction occurs due to the influence of a given current or potential [13,14]. Electrochemical detection is widely used in biosensor applications because of its low cost, simple construction, portability and ease of use. Electrochemical detection can be used to achieve low detection limits, either with or without sample preparation [15,16]. Nevertheless, electrochemical-biosensor platforms are still limited by the multiple steps involved in the testing process, including sample introduction, repetitive washing, and additional signaling-agent introduction. Further, a large sample volume is required, and the analysis is time-intensive [17]. Electrochemical measurements are grouped into four categories, i.e., voltammetry, potentiometry, impedance and conductometry. The grouping is based on changes in electrochemical properties detected during the biological attachment process [18].

The voltammetry method is the application of time-dependent potential to electrochemical cells. The function of the potential is to measure the resulting current. The results of voltammetry measurements are displayed in the form of a voltammogram. The principle of voltammetry measurement is based on measuring anode/cathode currents resulting from the oxidation/reduction reaction of an electroactive species at a selected potential window [19]. To obtain an electrochemical signal, it is common to use electroactive indicators such as ferricyanide ([Fe(CN)_6_]^3-/4-^) and hexaammineruthenium(III) chloride (Ru(NH_3_)_6_]^3-/4-^). Based on the potential scanned, the types of voltammetry are divided into cyclic voltammetry (CV), differential pulse voltammetry (DPV), square wave voltammetry (SWV), and linear sweep voltammetry (LSV) [20]. In the voltammetry method, the change in current resulting from electrochemical reduction or oxidation is monitored directly with time, while a constant potential is maintained at the working electrode in relation to the reference electrode [15]. The potentiometry method is an electroanalytic technique that detects ionic activity in samples [21]. Potential measurements are made when no current flows (*I* = 0) [13]. In the potentiometry method, two reference electrodes are used to measure the potential as it passes through the membrane and selectively reacts with the desired charged ion [15].

The impedance method generally describes an electrochemical analysis technique that, in its application, uses an alternating current or voltage (AC) in the system under investigation. This is followed by measuring the response in the form of AC current or voltage as a function of frequency [22]. During the experiment, the response of the system to the AC signal is recorded. This response is usually represented in a complex plane as impedance (*Z*), resistance value (*R*_s_), electron transfer resistance (*R*_ct_), double-layer capacitance (*C*_dl_) and angular frequency. The frequency can cover a wide spectrum, from very low frequencies (typically mHz) to high frequencies [23]. Electrochemical impedance measurement is carried out using a small excitation signal so that the cell response is pseudolinear [13].

The conductometry method is used to measure changes in the electrical conductivity of a solution or medium due to changes in the composition of the solution or medium during a chemical reaction. In its use, enzymes whose products are charged are used to produce changes in ionic strength, thereby increasing conductivity [15]. The advantages of using the conductometry method are the use of alternating voltages with low amplitudes that allow the Faraday process to be avoided at the electrodes, the use of a simple reference electrode, the low production costs and a high degree of integration with cheap film standards [24]. However, there is a drawback of the conductometric method: the ionic species produced must be able to change the total ionic strength significantly to obtain reliable measurements [13]. There are several methods that have been developed in the analysis of protein biomarkers of a disease, including gel electrophoresis, mass spectrometry and ELISA (enzyme-linked immunosorbent assay), but in their use, they require a lot of time, are expensive, and even require highly skilled human resources and other preparations [25]. For example, the cancer biomarker EpCAM (epithelial cell adhesion molecule) is generally detected using ELISA, PCR and cytometry, but researchers developed an electrochemical biosensor using rGO@TiO_2_ nanocomposites [26]. This artificial biosensor showed promising results for the detection of cancer biomarkers in serum samples, as shown by excellent electronic properties, selectivity and serves as a suitable sensing layer. So, it is believed that the biosensor created has the potential to be used in monitoring other cancer biomarkers.

## Protein biomarkers

Proteins are biological macromolecules in the body that play an important role in the metabolic catalysis process, transporting molecules across cells and cell apoptosis. Overexpression of a protein can be associated with certain types of pathogens [27]. Detecting biomarkers involves measuring the immune response and hormonal changes associated with a developing disease [28]. Biomarker proteins are only present at the molecular level during the early stages of a disease [27]. Protein biomarkers have been widely used in the field of applied research related to genomics and proteomics techniques [29-31]. Proteins are key compounds in different biological cells, tissues and organs [32]. Protein is a very informative type of biomarker, so many protein biomarkers are used in the application of clinical diagnosis and treatment of a disease. Biomarker proteins are expressed differently depending on the type of disease and provide various information about disorders that occur in the body. Different expressions of each biomarker protein can occur due to different protein processing in the body [33]. The research of Hartati *et al.* [34] reported using a gold bioconjugate modified electrochemical biosensor to detect epithelial sodium channel protein (ENaC), a protein biomarker of hypertension.

Molecular biomarkers are a broad scope for all biomarkers, both existing and to be discovered, and which can be measured or detected based on molecular characteristics (gene arrangement, proteomic analysis and complex multiplex analysis) and modified versions of the analytes [35]. According to the World Health Organization [36], biological markers are substances, processes, or structures in the body and can be measured to predict a disease. Apart from that, it can also be said to be a collection of certain molecules that can help with the diagnosis or prognosis of an abnormality in the body [37]. Biomarkers are used in clinical practice to provide different treatments or health care for each individual depending on the type of disease [38], so they are objective indications of medical conditions that can be observed accurately from outside the patient [39]. Generally, research or clinical practice using biomarkers can clinically predict a disorder or disease [40], demonstrate knowledge of clinical pharmacology, and provide a design basis for safe, rapid, and definitive clinical trials [41].

Determining biomarkers must be considered to obtain feasibility and ease of clinical use, so preclinical and clinical validation studies are needed first [39]. Biomarkers are classified into prognostic and diagnostic biomarkers. Prognostic biomarkers are related to disease recurrence information, while diagnostic biomarkers are related to the detection of a disease [12,42,43]. These biomarkers are used to predict the future clinical progression, severity, or risk of recurrence of a disease in a patient. Prognostic biomarkers play a crucial role in personalized medicine, allowing healthcare providers to tailor treatment plans to individual patients based on their predicted disease outcomes. Biomarkers have various functions, including the detection of disease, detection of abnormal conditions in the body (i.e., elevated blood glucose levels), monitoring of health status, and monitoring of clinical response to interventions (*e.g.*, blood cholesterol) [41].

Based on their characteristics, biomarkers are grouped into imaging biomarkers and molecular biomarkers. Imaging biomarkers are specific characteristics or features identified through medical imaging techniques that provide valuable information about the presence, progression, or severity of disease, as well as treatment response. Imaging biomarkers include positron emission tomography, magnetic resonance imaging, and computerized axial tomography. They enhance diagnostic accuracy, inform clinical decisions, and contribute to better patient outcomes. Meanwhile, the molecular biomarkers include proteins, DNA, RNA, small metabolites, and lipids [44]. Biomarkers are found in several biological fluids, such as serum and plasma, whole blood, sweat, nasal secretions, urine, sputum, lacrimal, bronchial, amniotic fluid, pleura, seminal fluid, and cerebrospinal fluid [33]. Protein biomarkers are of great interest because they can be detected in various analytical instruments to identify and measure proteins in complex biological samples [45]. The best use of biomarkers is demonstrated by tests that are accurate, easy to perform, and relatively non-invasive [46].

The following are several examples of protein biomarkers widely used for disease detection. Biomarker proteins in pancreatic cancer include KRAS (Kirsten Rat Sarcoma Viral Oncogene), MBD3L2 (Methyl-CpG Binding Domain Protein 3 Like 2), DPMI mRNAs (Dolichol phosphate mannose synthase), and ACRV1 (Acrosomal Vesicle Protein 1) [47]. Biomarkers for cervical cancer, namely Ki-67, BD ProEx C, and Cytoactiv HPV-L1 (Human Papillomavirus) [48]. In breast cancer, the most widely used biomarkers include HER2 (Human epidermal growth factor receptor 2), BRCA1 and BRCA2 (Breast cancer type 1 and 2 susceptibility proteins), CEA (Carcinoembryonic antigen), MUC1 (Mucin 1), VEGF (Vascular endothelial growth factor), CA15-3 (Cancer antigen 15-3), microRNA (miRNAs) [49]. The biomarkers for liver cancer are AFP (Alpha fetoprotein) and CEA, and the biomarker for prostate cancer is PSA (Prostate-specific antigen) [50]. The emergence of the novel SARS-CoV-2 disease in late 2019 has also led to the identification of numerous protein biomarkers associated with the disease, such as receptor-binding domain (RBD) protein, spike protein, and nucleocapsid protein (N protein).

## Biosensors detection mechanism of protein biomarker

The biosensor protein detection mechanism can be carried out in two steps, *i.e*., directly (label-free) and indirectly (labeled), depending on the output signal caused by the binding of the analyte to the labeled compound. Label-free detection is simpler, where antibodies are immobilized on the electrode surface, and changes in their electronic properties are immediately detected due to the formation of immunocomplexes [51]. In using the label-free detection technique, the signal response changes when the analyte molecule binds to the transducer surface. There are weaknesses in label-free detection, such as the occurrence of binding of non-analyte components to the sample matrix on the sensor surface, which affects false positive results. In label-free detection, it must be ensured that only the analyte bound to the appropriate biologically identifiable compound is immobilized on the electrode surface to obtain a significant change in signal response [52].

In labeled detection, further labeling of antibodies using enzymes or other molecules is required. In addition to enzymes, other labels are used, such as nanoparticles, fluorescent or electrochemiluminescent probes, and radionuclides [52]. Labeling is time-consuming, complex, has many steps and does not allow real-time detection and the use of labels can interfere with analyte binding, which can lead to distorted results [51]. The main advantage of the label detection method is that it has a higher potential for detection at lower concentrations. In addition, labeled detection can minimize false positive results because in labeled biosensors the final result is determined by the labeled compound whose binding is independent of the matrix [52].

### Detection of protein biomarkers in a label-free biosensor

**Figure 1.**
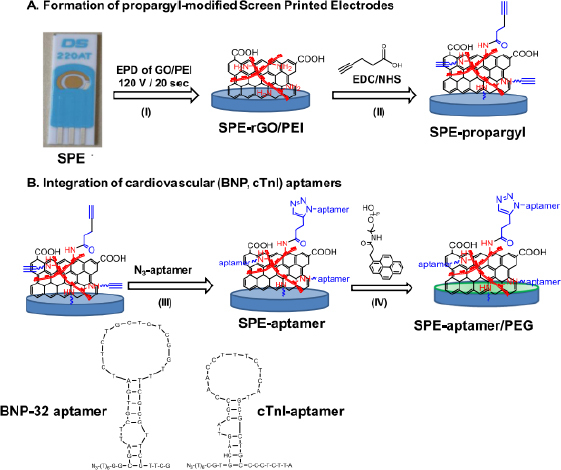
Schematic (A) surface modification of the gold SPE sensor by electrophoretic deposition of GO/PEI solution forms a thin layer of rGO/PEI; (B) integration of aptamers and passivation with synthetic pyrene-PEG (shown with green layer). Reproduction from [53] with copyright permission.

The following are several examples of label-free electrochemical biosensor research that has been conducted (shown in Table 1). Grabowska *et al*. [53] developed an aptamer-based electrochemical biosensor for early detection of cardiovascular disorders using two biomarkers, namely brain natriuretic peptide (BNP-32) and cardiac troponin I (cTnI). This study used commercial gold-based screen-printing electrodes (SPE) modified by electrophoretic deposition (EPD) using reduced polyethylenimine (PEI)/reduced graphene oxide (rGO) nanocomposite films, resulting in a robust and sensitive electrochemical platform for BNP-32 and cTnI sensing without the need for any labels (see Figure 1). The presence of the amine group on PEI facilitates the binding of the BNP-32 and cTnI aptamers via a propargylacetic acid linker followed by Cu(I)-based click chemical attachment to the azide and ending with the aptamer. Apart from that, modifications were also made by adding pyrene anchors carrying polyethylene glycol (PEG) units to ensure that the sensor is low in biofouling and has high specificity. Electrochemical measurements were carried out using differential pulse voltammetry with a [Fe(CN)_6_]^4−^ redox probe. This sensor has a detection limit of 0.9 pg/mL.

**Table 1.** application of label-free biosensors for detection of protein biomarkers of a disease in the last six years.

Working electrode	Bioreceptors	Protein biomarkers	Type of disease	Detection limit	Ref.
Gold electrode (GE)	D-fructose 6-phosphate (F6P)	Phosphoglucose isomerase from rabbit muscle (RmPGI)	Cancer in human plasma	6.6×10^-15^ M	[55]
GE	Synthetic Peptides PCT BP3	Procalcitonin (PCT)	Sepsis	12.5 ng/mL	[56]
Screen-printed gold electrode (SPGE)	Molecularly imprinted polymers (MIPs)	Hemeprotein myoglobin	Cardiovascular disease (CVD)	2.1×10^-3^ and 14×10^-3^ ng/mL	[57]
Printed circuit board (PCB)	BSA antibody	Bovine serum albumin (BSA)	Inflammation	2.89 ng/mL	[58]
Gold disk electrode (GDE)	Anti-tau antibody	Tau-441	Neurodegeneration	-	[59]
GE	EGFR antibody	EGFR antigen	Breast cancer	6.9×10^-3^ ng/mL	[60]
GE	TNF- α antibody	Tumor necrosis factor alpha (TNF- α) protein	Inflammation	10^-12^ M	[61]
GE	S100 beta antibody and CRP antibody	C-reactive proteins (CRP) and S100 beta proteins	CVD	10 ng/mL	[62]
ZnO	α-cTnT and α-cTnI antibody	Cardiac troponin T (cTnT) and cTnI	Myocardial infarction (MI)	10^-3^ ng/mL	[63]
ITO	Anti-EpCAM	EpCAM	Tumour	6.5×10^-3^ ng/mL	[26]
ITO	Anti-TNF-α	TNF-α	Cancer	1.39×10^-3^ ng/mL	[64]
GDE	TdT -mediated G-quadrupplex complex of 3’-OH terminal	Hemin	Diseases of thrombin	31×10^-13^ M	[65]
GE	Anti-IgG	Immunoglobulin G (IgG)	Inflammation	6×10^-18^ M	[66]
GE	Anti-CRP	CRP	CVD and Inflammatory diseases (an acute-phase protein)	2.25×10^-6^ ng/mL	[67]
GE	N-(5-phosphate-D-arabinoyl)-2-aminoethanamine (5PAED)	Autocrine motility factor-phosphoglucose isomerase (AMF-PGI)	Cancer	4.3×10^-14^ M	[68]
Gold-based SPE	BNP-32 aptamer and cTnI aptamer	Brain natriuretic peptide (BNP-32) and cTnI	CVD	0.9×10^-3^ ng/mL	[53]
Titanium foil	Cobalt-functionalized TiO_2_ nanotubes (Co-TNTs)	SARS-CoV-2 S-RBD protein	SARS-CoV-2	7×10^-10^ M	[69]
GE	Peptide ligan (H-C-acp-acp-FALGEA-NH_2_)	Glioblastoma (GBM)-derived exosomes	Glioblastoma - the most fatal tumors in the brain	7.83×10^3^ particle/μL	[70]
Screen-printed carbon electrode (SPCE)	Anti-ENaC antibody	ENaC protein	Salt-sensitive hypertension	0.198 ng/mL	[71]
GE	Antibody-tau-441	Tau-441	Dementia	4.6×10^-16^ M	[72]
Glassy carbon electrode (GCE)	pyrrole-3-carboxylic acid monomer	BRCA1 gene	Breast cancer	3×10^-15^ M	[73]
SPGE	Self-assembled monolayer (SAM) of cysteamine (CA)	α-amilase	Stress-related changes in the body	< 3.0×10^2^ ng/mL	[74]
Glass capillary	Molecularly imprinted polymers (MIPs)	Trypsin enzyme	Digestive disease	< 4.1×10 ng/mL	[75]
SPE	βHBA and NEFA antibodies	Β-hydroxybutyrate (βHBA) and non-esterified fatty acid (NEFA)	Dairy cow metabolic diseases	0.00011 M and 0.000111 M	[76]
GCE	IgE-aptamer	Immunoglobulin E (IgE)	Allergic reactions and parasitic diseases	4.2×10^-5^ ng/mL	[77]
Graphene oxide (GO)/gold nanoparticles (GNPs) hydrogel	Thiolated cellular prion protein (PrP^C^) peptide probe	Amyloid-beta oligomers (AβO)	Alzheimer’s disease	10^-13^ M	[78]
Au nanostructured gold disc electrode	Anti-rhuEPO Antibody	Recombinant human erythropoietin (rhuEPO)	Erythropoiesis (formation of erythrocytes in the bone marrow)	10^-12^ M	[79]
GCE	miRNA-21 aptamer	miRNA-21	Breast cancer	2.3×10^-15^ M	[80]
Glassy electrode	Anti-VEGF antibody	VEGF	Angiogenesis, vasculogenesis, and endothelial cell growth	81.46×10^-3^ ng/mL	[81]
SPCE	Anti-ENaC antibody	ENaC protein	Salt-senstive hypertension	0.037 ng/ml	[82]
GE	DGV peptide	DENV-2-NS1 protein	Dengue	1.49×10^-3^ ng/mL	[54]
Gold chip electrode	2008s aptamer	Plasmodium falciparum lactate dehydrogenase (PfLDH)	Malaria	8.4×10^-13^ M	[83]
GE	Anti-NUMA1 antibody and anti-CFHR1 antibody	Nuclear mitotic apparatus protein 1 (NUMA1) and complement factor H-related 1 (CFHR1)	Bladder cancer	1.29 ng/mL and 0.97 ng/mL	[84]
GCE	Anti-CA15-3	CA15-3	Breast cancer	0.32 mU/mL	[85]
Gold interdigitated micro-electrode arrays (IDμE)	Anti-HER4 affimer	HER4	Tumour	< 10^-12^ M	[86]
Platinum	Anti-PARK7/DJ-1 antibody	Parkinson’s disease protein 7/protein deglycase DJ-1 (PARK7/DJ1)	Parkinson’s disease	7.5 ng/mL	[87]
GCE	Spike SARS-CoV-2 antibody	Spike protein SARS-CoV-2 antigen	SARS-CoV-2	10^-11^ ng/mL	[88]
SPCE	Anti-EnaC antibody	ENaC protein	Hypertension	8.4×10^-2^ ng/mL	[34]
GE	Anti-VEGF antibody (VEGFab) and anti-PSA antibody (PSAab)	VEGF and PSA	Prostate cancer (PCa)	50 pg/mL and 1 ng/mL	[89]
GDE	Self-assembled monolayer from 11-ferrocenyl-undecanethiol (11FcC) and polyethylene glycol (PEG) containing the thiol (PEG thiol)	Human prostatic acid phosphatase (hPAP)	PCa	1.119×10^-11^ M	[90]
GE	NGAL peptide	Neutrophil gelatinase-associated lipocalin (NGAL)	Acute kidney injury and the diabetic	3.93 ng/mL (SWV) and 1.74 ng/mL (EIS)	[91]
Multiwall carbon nanotube (MWCNT) electrodes	Anti-OV6-Ab	OV6 marker	Cancer	-	[92]
Au micro-gap electrode	Bioprobe DNA 3 way-junction (3WJ)	cTnI	CVD	10^-12^ M	[93]
GE	HER2-specific hybrid aptamer-polyclonal antibody and antibody-based sandwich	HER2	Breast cancer	1 ng/mL	[94]
GE	Dual-functional hairpin dNA probe which consists miR-16 complementary sequence and AFP aptamer sequence	miRNA-16 and AFP	HCC	1.4×10^-10^ M	[95]
Carbon electrode	Anti-lysozyme aptamer	Lysozyme	Breast Cancer, alzheimer’s, malaria	90 ng/mL	[96]
3D nanoprinted gold micropillar array electrode	SARS-CoV-2 spike RBD protein	Anti-spike antibodies CR3022	SARS-CoV-2	0.4 BAU/mL	[97]
ITO	Anti-Aβ42	Aβ42	Alzheimer	3.7×10^-4^ ng/mL	[98]
316 L stainless-steel plate electrode	Gelsolin-actin	Lysophosphatidic acid (LPA)	Ovarian cancer	7×10^-7^ M	[99]
ITO micro-electrode array	Anti-plasma phosph-orylated-tau threonine 181 (p-tau181) antibodies	Plasma phosphorrylated-tau threonine 181 (p-tau181)	Alzheimer and mild cognitive impairment (MCI)	9.2×10^-7^ ng/mL	[100]
MGCE modified Mg_0.5_Cu_0.5_Fe_2_O_4_-Au	DNA-aptamer	CA125	Ovarian cancer	4.4 U/mL	[101]
FTO electrode modified graphene oxide (GO) decorated with gold nano-flower nanostructures (GO@Au-NS)	Thiolated DNA capture probe against miRNA-223 (Cap-223)	miRNA-223	Colorectal cancer	1.2×10^-20^ M	[102]
GE modified gold nanoparticles-black phosphorus (AuNPs@BP@PDA)	Synthetic peptide receptor (C-terminus incorporated to gold binding peptide (GBP)	CRP	Crohn’s disease	0.7 ng/mL	[103]
α-Fe_2_O_3_/carbon cloth yarn	Anti-IL-6 antibodies	Interleukin-6 (IL-6)	Cancer	2.6×10^-4^ ng/mL	[104]
Magnetic glassy carbon electrode (MGCE)	Peptide nucleic acid (PNA)	TP53 gene	Tumour, cancer	2.6×10^-13^ M	[105]
Gold-interdigitated microelectrodes (IdμEs) modified VS_2_	MMP-9 antibody	MMP-9 antigen	Ocular inflammatory	1.344×10^-9^ ng/mL	[106]
GE	HRP-conjugated antibody of telomerase	Telomerase antigen	Cancer	0.078 IU/mL	[107]
ITO	Anti-CYFRA 21-1	Cytokeratin subunit 19 (CYFRA 21-1)	Lung cancer	4.7×10^-6^ ng/mL	[108]
ITO	Anti-SP17 antibodies	Sperm protein-17 (SP17)	Cancer	47.57×10^-3^ ng/mL	[109]
ITO modified AuNPs/Ti_3_C_2_-mxenes	cTnI-specific aptamer (SH-Apt_cTnI_)	cTnI	Acute myocardial infarction	1.4×10^-7^ ng/mL	[110]
GO/ amino substituted polypyrrole polymer modified disposable electrode	Anti-CALR antibodies	Calreticulin (CALR)	Cancer	10.4×10^-6^ ng/mL	[111]
SPCE modified AuNPs/GO-COOH	CRP aptamer probes	CRP	CVD and inflammation	0.001 ng/mL	[112]
GCE modified PtNi nanocubes assemblies	HE4 antibody (HE4-Ab)	Human epididymis protein 4 (HE4)	Epithelial ovarian cancer (EOC)	0.11×10^-3^ ng/mL	[113]
GE	Cis P-tau monoclonal antibody (mAb)	Cis phosphorylated tau (cis P-tau)	Alzheimer	2×10^-14^ M	[114]
SPCE	Aptamer ENaC	ENaC protein	Salt-sensitive hypertension	0.012 ng/mL	[115]
SPCE	Anti-ENaC antibody	ENaC protein	Salt-sensitive hypertension	0.113 ng/mL	[116]
SPCE modified Pd	Antibodies specific to HER2 (anti-HER2)	HER2	Breast cancer	1 ng/mL	[117]
GCE modified carbon nanofiber	Aptamer	Cytochrome c (Cyt c)	Cancer	7.4×10^-10^ M	[118]
GE	Aptamer-based specific recognition with CRISPR-Cas12a	SARS-CoV-2 nucleocapsid antigen	SARS-CoV 2	0.077 ng/mL	[119]
GDE	Anti-MCM5	Mini chromosome maintenance protein 5, MCM5	Cervical cancer	2.9×10^-12^ M	[120]
SPCE	Anti-ENaC antibody	ENaC protein	Salt-sensitive hypertension	0.0372 ng/mL	[116]
SPCE	Bicyclic peptides	Human urokinase-type plasminogen activator (h-uPA)	Cancer	9 ng/mL	[121]
SPCE	Anti-ENaC antibody	ENaC protein	Salt-sensitive hypertension	0.110 ng/mL	[122]

Jalil *et al.* [26] developed a label-free electrochemical biosensor for early detection of a tumor biomarker EpCAM. This is the first research study regarding the creation of a transducer platform based on rGO@TiO_2_ nanocomposites for determining cancer biomarkers. In this study, antibodies (anti-EpCAM) were immobilized directly on the surface of the rGO@TiO_2_/indium tin oxide (ITO) electrode and were ready to capture the EpCAM antigen. The development of the biosensor was carried out using reduced graphene oxide (rGO) modified with titanium dioxide (TiO_2_) nanoparticles to form rGO@TiO_2_ nanocomposites, which were synthesized through a hydrothermal process. The rGO@TiO_2_ nanocomposite was deposited on an ITO-coated glass substrate by electrophoretic deposition method so that the modification became rGO@TiO_2_/ITO electrode. Spectroscopy techniques, microscopic identification and electrochemical measurements were used to determine the success of the deposition stage. After the electrodes were modified, they were used for covalent immobilization of the EpCAM monoclonal antibody (anti-EpCAM/rGO@TiO_2_/ITO electrode). After modification was completed, EpCAM was immobilized at the electrodes. Bovine serum albumin was used as a blocking agent to avoid non-specific binding of EpCAM. Electrochemical measurements were carried out using DPV and EIS with the electroactive indicator ferricyanide ([Fe(CN)_6_]^3-/4-^). The detection range is 0.01-60 ng/mL, with a detection limit of 0.0065 ng/mL.

**Figure 2.**
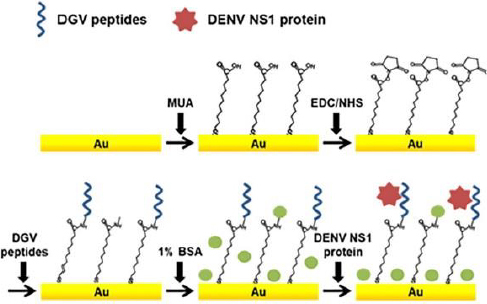
label-free biosensor schematic for detection of DENV NS1 protein. Reproduction from [54] with copyright permission.

In addition, Kim *et al.* [54] also developed a label-free electrochemical biosensor for the detection of nonstructural dengue virus protein (DENV) 1 (NS1), which is a specific and sensitive biomarker for the diagnosis of dengue fever (shown in Figure 2). In this research, a series of synthetic peptides substituted with amino acids was designed. This synthetic peptide acts as a recognition compound that will recognize the DENV-NS1 target. Five synthetic peptide derivatives (DGV BP1, BP2, BP3, BP4 and BP5) were used, rationally designed and chemically synthesized. Modification of the biosensor was carried out with an Au substrate prepared by evaporation of gold on a clean silicon wafer, then coated with titanium. The gold substrate was placed in a piranha solution to remove residual substances, which was washed with distilled water. Then, the Au substrate was dried under nitrogen flow and immersed in an ethanol solution of 1-mercaptodecanoic acid (MUA) overnight. The activated gold substrate was dried under a nitrogen stream. MUA-activated Au substrates were immersed in ethyl(dimethylaminopropyl)carbodiimide (EDC) and N-hydroxysuccinimide (NHS) solutions in methanol. The active Au substrate was rinsed using methanol and immersed in PBS. After functionalization of the Au substrate, electrode assembly was carried out, and the synthetic peptide was dropped on the surface of the Au substrate. Synthetic peptides are covalently immobilized onto the gold sensor surface. The biomarker protein DENV-NS1 was dripped onto the surface of the modified electrode. In practice, the performance of the biosensors is monitored using SWV and EIS. Electrochemical analysis was carried out using PBS solution containing ferro/ferricyanide. The detection limit for NS1 was 1.49 g/mL.

### Detection of protein biomarkers by labelled biosensor

**Figure 3.**
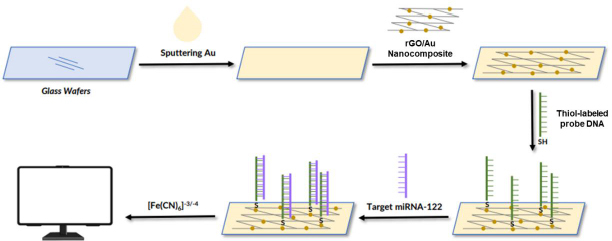
schematic of a thiol-labeled biosensor for the detection of miRNA-122 protein. Redraw from [123].

The following are several examples of the development of labeled electrochemical biosensors for the detection of protein biomarkers of diseases. Kasturi *et al.* [123] developed an electrochemical biosensor using a thiol-labeled probe DNA to detect microRNA-122 (miRNA-122), which is a biomarker of liver diseases, including hepatocellular carcinoma (HCC). This study developed an easy, effective, and sensitive RNA electrochemical biosensor for the detection of Au-loaded reduced graphene oxide (rGO) miRNA-122 synthesized by a simple hydrothermal reflux method. The thiol-labeled DNA probe was anchored at the rGO/Au nanocomposite binding site and recognized the target miRNA-122. This biosensor is shown in Figure 3. The rGo/Au nanocomposite serves to improve the performance of the biosensor due to the significant electron conductivity of the electrochemical surface area. Modification of the biosensor was carried out with a glass wafer. Gold (Au) was sprayed on the glass wafer by a sputtering system.

The Au-sputtered glass wafers were then cleaned with deionized water and dried under a nitrogen stream. Furthermore, the surface of the Au-modified glass wafer was dripped with rGO/Au nanocomposite. After the electrode surface was modified by the rGO/Au nanocomposite, the thiol-labeled DNA probe solution in tris-EDTA was dispersed on the electrode surface and incubated for 12 h under moist conditions. Then, BSA 1 % solution was used to block the electrode surface. Next, the target miRNA in the Tris-EDTA solution dripped on the surface of the electrode, where the probe DNA was immobilized. The thiol was used as a linker to label the DNA probe solution dispersed on the surface of the rGO nanocomposite modified Au electrode to identify miRNA-122 targets. Electrochemical measurements were carried out using CV and DPV using electrolyte solution [Fe(CN)_6_]^-3/-4^ to determine the success of immobilizing DNA probes and target miRNA hybridization. This biosensor showed a linear response for various target concentrations of miRNA-122 in the range of 10 μM to 10 pM with a detection limit of 1.73 pM.

Muñoz-San Martín *et al.* [124] developed an electrochemical peptide biosensor based on the on-off method used for the detection of pancreatic cancer using a biomarker in the form of trypsin, which belongs to the protease family. Double-labeled short synthetic peptides were used in this biosensor modification. Double labeling was performed using fluorescein isothiocyanate (FITC) and biotin. The biosensor development strategy was carried out using electrodes in the form of an SPCE modified using magnetic microbeads (MBs). The MBs surface was double-labeled with FITC immobilization, and a biotin-labeled peptide probe immobilized through the biotinylated end of the neutravidin-MBs surface. MBs are used to support the performance of biosensors with lower non-specific adsorption capabilities and higher affinity for biotin. Further cleavage is carried out using a target enzyme (trypsin) that cleaves the peptide at the C-terminal end of arginine so that the FITC-bound peptide moiety is released from the MBs. Enzymatic labeling was performed using anti-FITC conjugated with horseradish peroxidase (HRP) to (HRP-anti-FITC). The modified MBs were magnetically captured on the surface of the SPCE, thus modifying the biosensor to become HRP-anti-FITC/Peptide/Neutravidin-MBs/SPCE. In the development of this biosensor, an on-off approach was used due to the lower amount of HRP-anti-FITC, which will attach to the missing peptide fragment that carries the FITC moiety from the surface of MBs. Electrochemical measurements were carried out using the amperometry method with a redox medium in the form of hydroquinone (HQ) and the enzymatic substrate H_2_O_2_. The sample used for trypsin detection is human cell lysate. The high sensitivity of the biosensor can determine trypsin in clinical samples and quantify the trypsin content in cell lysates with the ability to differentiate between pancreatic and non-pancreatic cancer cells. The results of the biosensor showed a detection limit of 0.16 g/mL.

Recently, researchers have increasingly focused on simultaneous biomarker detection, enabling the analysis of multiple analytes in a single assay. This approach offers several advantageous features, addressing the limitations associated with single-analyte detection methods and catering to the need for comprehensive and efficient analysis. Simultaneous biomarker detection is particularly valuable when faced with limited sample volumes and contributes to more accurate diagnoses. Anabalagan *et al.* [125] conducted a study that exemplifies this trend. They simultaneously developed an innovative approach for detecting two cancer biomarkers, CEA and AFP, through the design of two distinct redox-labelled detection probes. Specifically, silver NPs functionalized with CEAAb2 and 1-amino anthraquinone were employed for CEA detection, while polyaniline NPs were functionalized with ferrocenecarboxaldehyde (Fc-CHO) for detecting AFP. The detection process involved applying different voltage pulses in a sequence, including 0 V for 10 s, -0.75 V (potential AQ) for 10 s, 0 V for 10 s, and +0.5 V (Potential Fc) for 10 s, hence exhibited excellent sensitivity, specificity, and minimal cross-reactivity between the two targeted biomarkers, with detection limits of 30 pg/mL for AFP and 80 pg/mL for CEA. Moreover, the proposed sensor was used to determine APF and CEA in human blood serum. In addition to several examples already described, Table 2 shows other examples of the use of labeled biosensors for protein biomarker detection.

**Table 2.** application of labeled biosensor for detection of protein biomarkers of disease in the last six years.

Working electrode	Bio-receptors	Protein biomarkers	Type of disease	Detection method	Detection limit	Ref.
GE	DNA Probe	miRNA-375, miRNA-141, and PSA	PCa	Methyl blue labeled	miRNA-141: 8×10^-10^ M, miRNA-375: 8×10^-10^ nM, PSA: 10^-12^ M	[126]
GE	HER2 antibody	HER2	Breast cancer	Nanoprobe catalytic labeled	10^-5^ ng/mL	[25]
GE	Probe DNA	miRNA-122	Liver diseases including HCC	Thiol-labeled	1.73×10^-12^ M	[123]
GE	DNA Probe	DNA H1-MB and H2-MB sample	Liver disease	Methylen blue labeled	4.1×10^-5^ ng/mL	[127]
GE	Anti-TNF-α antibody	Protein TNF-α	Inflammation	Biotin labeled	11.21×10^-3^ ng/mL	[128]
SPE	Heparin (Hep) from Hep-Au@Fe_3_O_4_	Eosinophil cationic protein	Asthma	Heparin labeled	3×10^-10^ M	[129]
SPCE	HRP-anti-FITC	Trypsin	Cancer	Fluorescein isothiocyanate (FITC) and biotin labeled	160 ng/mL	[124]
GCE	AβO-specific aptamer	AβOs	Alzheimer's disease	Thiol labeled	1.22×10^-3^ ng/mL	[130]
GCE	Catalytic hairpin assembly (CHA)	miRNA-1246 and miRNA-4521	Hemophilia	QDs@ZIF-8 labeled	miRNA-1246: 1.9×10^-16^ M miRNA-4521: 2.8×10^-16^ M	[131]
GE	ssDNA aptamer	Transforming growth factor b1 (TGF-b1)	HPV-16 and parovovirus B19 (PB-19)	N-succinimidyl S-acetylthioacetat Labeled	2×10^-10^ M	[132]
Gold nanostructured electrodes	Anti-OTOL1 Dan anti-PRES	Otolin-1 and prestin proteins	Hearing disorders	Methylen blue labeled	-	[133]
GE	Probe sequence	miRNA-155	Breast cancer	Polyethyleneimine-silver nanoparticles (PEI-Ag NPs)	20 zmol	[134]
GE	Tetrahedral DNA nanostructure (TDNs)-aptamer	HER2	Breast cancer	Horseradish peroxidase-labeled	0.08 ng/mL	[135]
GE	Apt15 and Apt29 aptamer	Thrombin	Hemostasis	Ferrocene labeled	7.6×10^-13^ M	[136]
GE	Anti-CA 15-3 monoclonal antibody	CA15-3	Breast cancer	Magnetic beads labeled	15×10^-6^ U/mL	[137]
GE	Biotin-DNA-biotin	miRNAs	Cancer	Enzyme labeled	10^-17^ M	[138]
SPCE	Anti-AFP	AFP	Liver cancer	Methylene blue labeled	8.5×10^-5^ ng/mL	[139]
ITO	Anti CA125 antibodies	CA125	Ovarian cancer	Silver@polypyrrole (Ag@PPy) labeled	10^-7^ ng/mL	[140]
SPCE	S9.6 antibodies (one anti-DNA/RNA antibody)	multiple miRNA biomarkers (miRNA-21, miRNA-155 and miRNA-10b)	Cancer	titanium phosphate nanospheres with different heavy metal ions (zinc, cadmium, lead),	1.3×10^-16^ M, 1.9×10^-16^ M, and 2.3×10^-16^ M	[141]
SPGE	Antibodies against HER-1 and HER-2	HER-1 and HER-2	Breast cancer	Horseradish peroxidase-labeled	1.06 ng/mL and 0.95 ng/mL	[142]
SPCE	CEA antibody and AFP antibody	CEA and AFP	Cancer	Silver nanoparticles and anthraquinone for CEA; and ferrocene for AFP	8×10^-2^ ng/mL for CEA and 3×10^-2^ ng/mL for AFP	[125]
GCE	Anti-miRNA-141 complementary sequence (ACP--141) and nti-miRNA-21 DNA probe	miRNA-141.and miRNA-21	Lung cancer	Methylene blue and ferrocene	8.9×10^-16^ M for miRNA-141 and 1.24×10^-15^ M for miRNA-21	[143]
GE	CA199 antibody	Carbohydrate antigen-199	Pancreatic cancer	Glucose oxidase-amino magnetic nanoparticles (AMNP) and gold- horseradish peroxidase	0.2 U/mL	[144]
Graphene/SPCE	N protein SARS-CoV-2	IgG-SARS-CoV-2 nucleocapsid	SARS-CoV2	Secondary antibody labeled with horseradish peroxidase	1:4947 v/v	[145]
GCE	g-C_3_N_4_/Fe_3_O_4_/ /aptamer	CA15-3	Breast cancer	Methylene blue-labeled	0.2 U/mL	[3]
SPCE	Human eukaryotic myelin basic protein (MBP)	anti-MBP	Multiple sclerosis autoimmune disease	Secondary antibody labelled with horseradish peroxidase (HRP-anti-hIgG)	0.016 ng/mL	[146]

## Future perspectives

Early detection of a disease can help control the infection of a disease more effectively so that it can treat patients on time [147]. The development of biosensor technology in the future will develop rapidly as the use of biosensors increases as a device for monitoring a person's health status. Biosensors are increasingly in demand due to their wide use in healthcare and medicinal applications, paving the way for better development [148]. A broad strategy for developing biosensors for protein biomarker detection can be carried out by establishing an economical, straightforward, reusable biosensor construction that has the potential for large-scale manufacture and rapid operation of biosensors. In the future, this strategy can be applied in the development of biosensors in general and can be used for the detection of various protein biomarkers of disease [75].

Nowadays, label-free biosensor detection techniques have made advances in the use of newer signal detection schemes. The use of nanotechnology-based transducers allows label-free biosensors to have high sensitivity, little analyte damage, and use little sample. Label-free biosensor techniques have excellent potential to meet the demand for higher-quality biosensors and have been widely developed over the last few years [149]. So far, the technical use of label detection in biosensors has increased detection potential at lower concentrations. However, the use of labeled compounds usually has high operational costs and longer testing times. In addition, real-time analysis is not possible, and the use of labels can disrupt the binding of analytes, causing distorted results [52]. The rapid development of biosensors in the future depends on the innovation of researchers to accept the opportunities and challenges in the development of electrochemical biosensors. The fields of electrochemistry, proteomics and biotechnology that continue to develop will have an impact on the development of reliable electrochemical biosensors in the diagnosis of protein biomarkers of a disease on the spot [47]. Electrochemical biosensors will grow yearly as a reliable analytical tool [150].

## Conclusions

Protein biomarkers are useful in the clinical detection of disease and monitoring health status that can indicate abnormal conditions in the body. Electrochemical biosensors have been widely used for early diagnosis applications of disease in recent years. Using electrochemical biosensors with simple construction, low cost, easy to use, portability, and low detection limits makes biosensors an alternative method for early detection of a disease. Label-free or labeled detection techniques on biosensors can be used according to research needs and requirements, such as the biomolecular compound used, the type of analyte and its biological binding site, biosensor construction, sample volume, operational costs, analysis time, and the desired detection limit and effectiveness of the use of biosensors.
